# A guidance system for robotic welding based on an improved YOLOv5 algorithm with a RealSense depth camera

**DOI:** 10.1038/s41598-023-48318-8

**Published:** 2023-12-02

**Authors:** Maoyong Li, Jiqiang Huang, Long Xue, Ruiying Zhang

**Affiliations:** Beijing Key Laboratory of Opto-Electromechanical Equipment Technology, Beijing Institute of Petrochemical Technology, Beijing, 102617 China

**Keywords:** Design, synthesis and processing, Mechanical engineering

## Abstract

Vision-based automatic welding guidance technology plays an essential role in robotic welding. A laser vision sensor (LVS) relies on manual intervention to guide the robot when near the workpiece, which reduces the autonomy of the welding robot and productivity. To solve this problem, a robot welding guidance system based on an improved YOLOv5 algorithm with a RealSense Depth Camera was proposed. A coordinate attention (CA) module was embedded in the original YOLOv5 algorithm to improve the accuracy of weld groove detection. The center of the predicted frame of the weld groove in the pixel plane was combined with the depth information acquired by a RealSense depth camera to calculate the actual position of the weld groove. Subsequently, the robot was guided to approach and move over the workpiece. Then, the LVS was used to guide the welding torch installed at the end of the robot to move along the centerline of the weld groove and complete welding tasks. The feasibility of the proposed method was verified by experiments. The maximum error was 2.9 mm in guiding experiments conducted with a distance of 300 mm between the depth camera and the workpiece. The percentage error was within 2% in guidance experiments conducted with distances from 0.3 to 2 m. The system combines the advantages of the depth camera for accurate positioning within a large field and the LVS for high accuracy. Once the position of the weld groove of the workpiece to be welded has been determined, the LVS combined with the robot can easily track the weld groove and realize the welding operation without manual intervention.

## Introduction

Welding in industrial manufacturing relies on mostly manual labor, leading to high labor intensity and low work efficiency. As populations age and welding environments become harsher, a shortage of skilled welders is becoming more common^1^. To overcome these challenges, the utilization of welding robots is on the rise in industrial production, enhancing both welding efficiency and precision^2^.

In real welding, scenarios such as randomly placed welding workpieces and unstable fixtures are common. This can lead to greatly differing weld positions, and both the welding robot teaching-playback mode and offline programming will face very large work^3^. Weld tracking technology has emerged as a solution to the problems caused by nonstandard workpieces^4^. A laser vision sensor (LVS) is typically installed ahead of the welding gun along the movement direction, and the weld seam information is acquired in advance through a sensor. Due to limitations in recognition distance and range, manual presetting of the trajectory or positioning of the sensor above the workpiece may be necessary for the sensor to detect the weld seam. While LVSs have yielded positive results in weld inspection, they are primarily suitable for a limited field of view and do not reliably determine the workpiece's position in large work areas.Therefore, an LVS is suitable for tracking a weld seam but not for determining the overall position of the weld seam^5^. Welding robots often still require manual intervention when welding nonconforming workpieces with uncertain positions.

Machine vision technology has been used for robotic weld seam identification. Weld seam identification is the key technology to automatically identify weld grooves and guide the robot along the groove ^6^. Using machine vision methods, researchers have achieved initial point detection of weld seams^7^, real-time weld seam tracking^8^, etc. Lan et al.^9^ used a 2D camera and a 3D laser vision sensor composite sensing method to identify and position harbor crane lugs and guide welding routes with precision and fine-scale guidance. Zhen et al.^10^ proposed an image processing method based on a priori knowledge to acquire feature points with subpixel accuracy in real time and a new online planning model of welding trajectories that automatically guided welding by robots without teaching. Wu et al.^11^ established a no-teaching position guidance method combining LVS position detection with externally directed motion data transmitted bidirectionally at high speed, which guided welding robots to move along complex trajectories. Mitchell et al.^12^ presented an algorithm for detecting welds at butt joints with an arc welding robot using computer vision. The method automatically subtracts the background from images obtained by a robot-mounted camera system. It can detect straight and curved welds without prior knowledge of the weld position. Radovan et al.^13^ proposed an algorithm based on template matching to identify and detect a weld's initial point, and the results showed that the algorithm could achieve a positioning accuracy of ± 0.5 mm for simple welds. Chen et al.^14^ proposed a feature point location method with only two contour scans that can effectively calculate the initial position of a weld seam. H.C. Nguyen et al.^15^ proposed an extraction algorithm using sliding vectors to find weld corner points that is a fast and reliable method for detecting laser stripe contour feature points. The conventional methods mentioned above overwhelmingly use graphical features, such as shape and size, which are detected based on a priori knowledge of an object in a more stable state in terms of its feature information.

The emergence of deep learning has greatly facilitated the development of computer vision. Deep learning is applied to a variety of vision fields, such as face detection^16^, medical lesion image segmentation^17^, and autonomous driving^18^. Deep learning techniques are also applied to detect and track weld seams. Du et al.^19^ used fast image segmentation, feature region identification using a convolutional neural network (CNN), and feature search techniques to accurately identify weld features for problems such as the presence of many very noisy images in gas-shielded welding. Xiao et al.^20^ proposed an adaptive feature extraction algorithm based on LVSs. Based on a laser streak image, typical welds are classified as continuous or discontinuous welds. A Faster R-CNN model is trained to identify the weld seam type and automatically locate the laser streak region of interest (ROI). Initial weld points are determined through point cloud processing before welding to achieve weld guidance. Jin et al.^21^ proposed a Mask R-CNN network-based a weld seam recognition model that uses migration learning to recognize weld seams in example images and segmentation, and the method can effectively identify complex weld seams to obtain accurate weld joint locations. Zhou et al.^22^ proposed a multifeature combination network in a single shot multibox detector (SSD) object detection framework with the characteristics of a weld seam detection task. This network merges the local and global information carried by multilayer features for weld seam detection and rapidly and accurately detects of weld seams. Yang et al.^23^ designed a lightweight multilayer CNN to detect weld groove edges that are disturbed by noise. The network is capable of extracting multilayer features, improving the resolution of weld groove edge detection with strong interference immunity. There are two main problems with the deep learning-based weld detection and localization methods mentioned above: the detection window is small and does not consider the detection and localization of weld seams in a large complex field, and the weld position detected by the algorithm is described by the 2D coordinates of the image, not the real coordinates of the weld in the global environment.

To enable a robot to acquire the real coordinates of a weld seam in a complex global environment and approach the seam autonomously for subsequent welding work, a welding robot guidance system combining deep learning and a depth camera was developed. The main contributions of this paper include the following. First, an improved You Only Look Once (YOLO) v5 weld groove detection algorithm is proposed by adding a coordinate attention module to YOLOv5. This algorithm can better focus on the weld groove features in complex environments. Then, an object detection algorithm is combined with a depth camera ranging algorithm, which can inspect the 3D coordinates of the weld groove in real-time with limited computing resources. The algorithm was deployed on a host computer for welding robot guiding experiments, and the results showed sufficient accuracy for welding robot guidance and subsequent welding in a global complex environment.

## Materials and methods

### Welding robot guidance system

The proposed robot welding guidance system based on an improved YOLOv5 with a RealSense depth camera, consists of a host computer and a welding robot.

**Figure 1:**
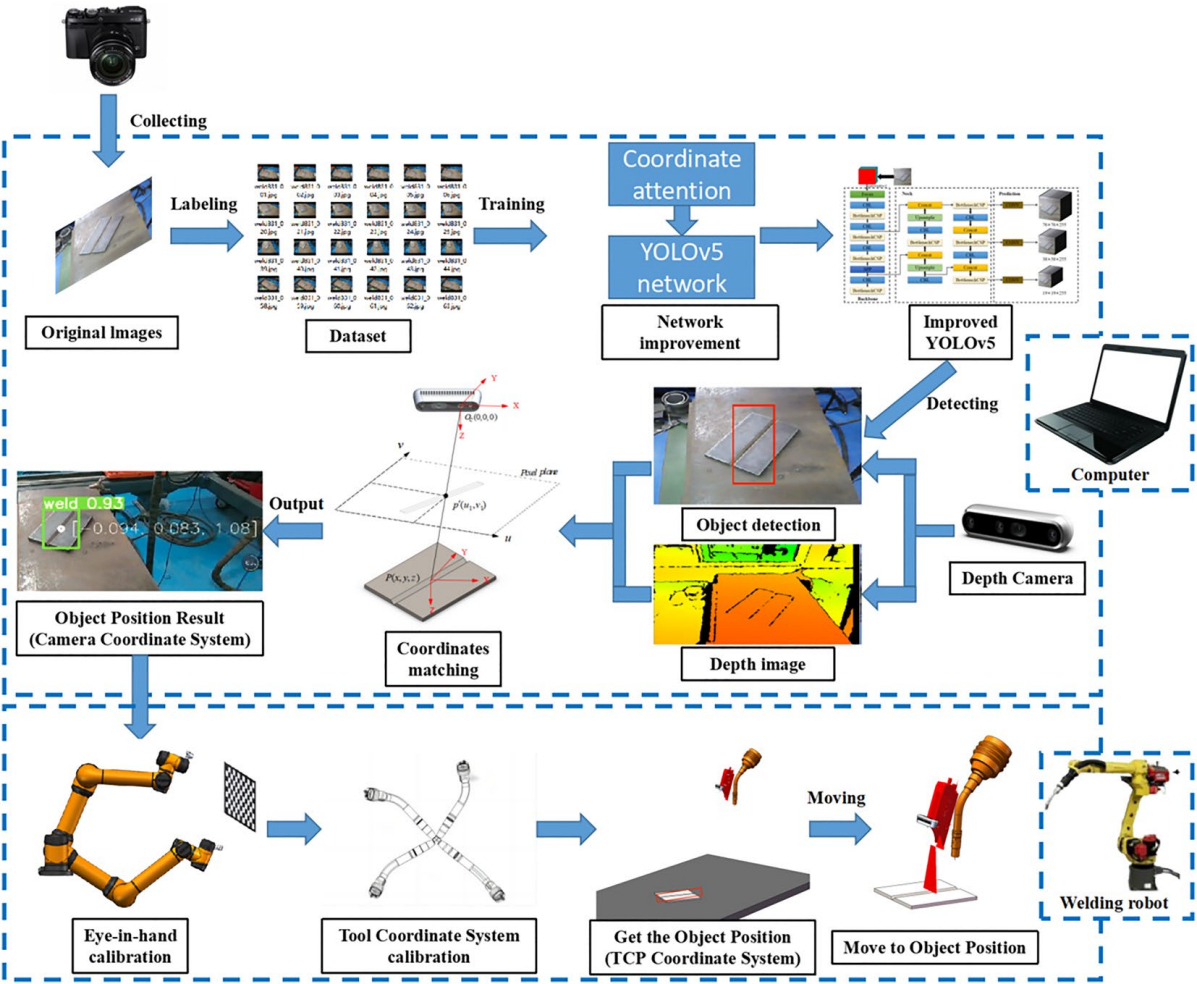
Flowchart of the detection and location.

The host computer processes the captured photo information to obtain the 3D coordinates of the objects and transmit them to the welding robot. The welding robot receives the coordinates and moves to the object position. The specific steps carried out by this system are as follows: First, a weld groove dataset is established and used to train a YOLOv5 object detection model to realize the intelligent recognition of weld grooves. Then, a depth camera acquires RGB images, which are used as input for the YOLOv5 object detection algorithm, and the model outputs the type and location of the weld groove. Third, the center points of the weld groove on the pixel plane are obtained according to the prediction frame, and the actual weld groove position is calculated using the 3D point cloud information obtained by the depth camera. Finally, through robot eye-in-hand calibration and tool center point (TCP) calibration, the 3D coordinates of the object in the robot TCP coordinate system are obtained to guide the robot toward the weld groove area. Figure 1 shows a flow chart of this system.

### Weld groove detection based on an improved YOLO v5 model

Object detection based on deep learning is mainly divided into two categories: two-stage object detection algorithms and one-stage object detection algorithms. Two-stage methods need to generate candidate detection regions before outputting results, which improves accuracy while reducing recognition speed, with representative algorithms including R-CNN^24^, Fast R-CNN^25^ and Faster R-CNN^26^. A one-stage algorithm is used to obtain the type of object and its coordinate position directly through the detection network, and typical methods include SSD^27^ and YOLO^28^. One-stage methods sacrifice only a small amount of accuracy in exchange for a significant increase in detection speed, making them widely used in the industry. YOLO is a One-Stage algorithm based on fully convolutional neural networks presented and proposed by Joseph Redmon et al. at CVPR in 2016, and 5 versions have been developed. In summary, the one-stage YOLOv5 algorithm is chosen for the system proposed in this paper.

**Figure 2:**
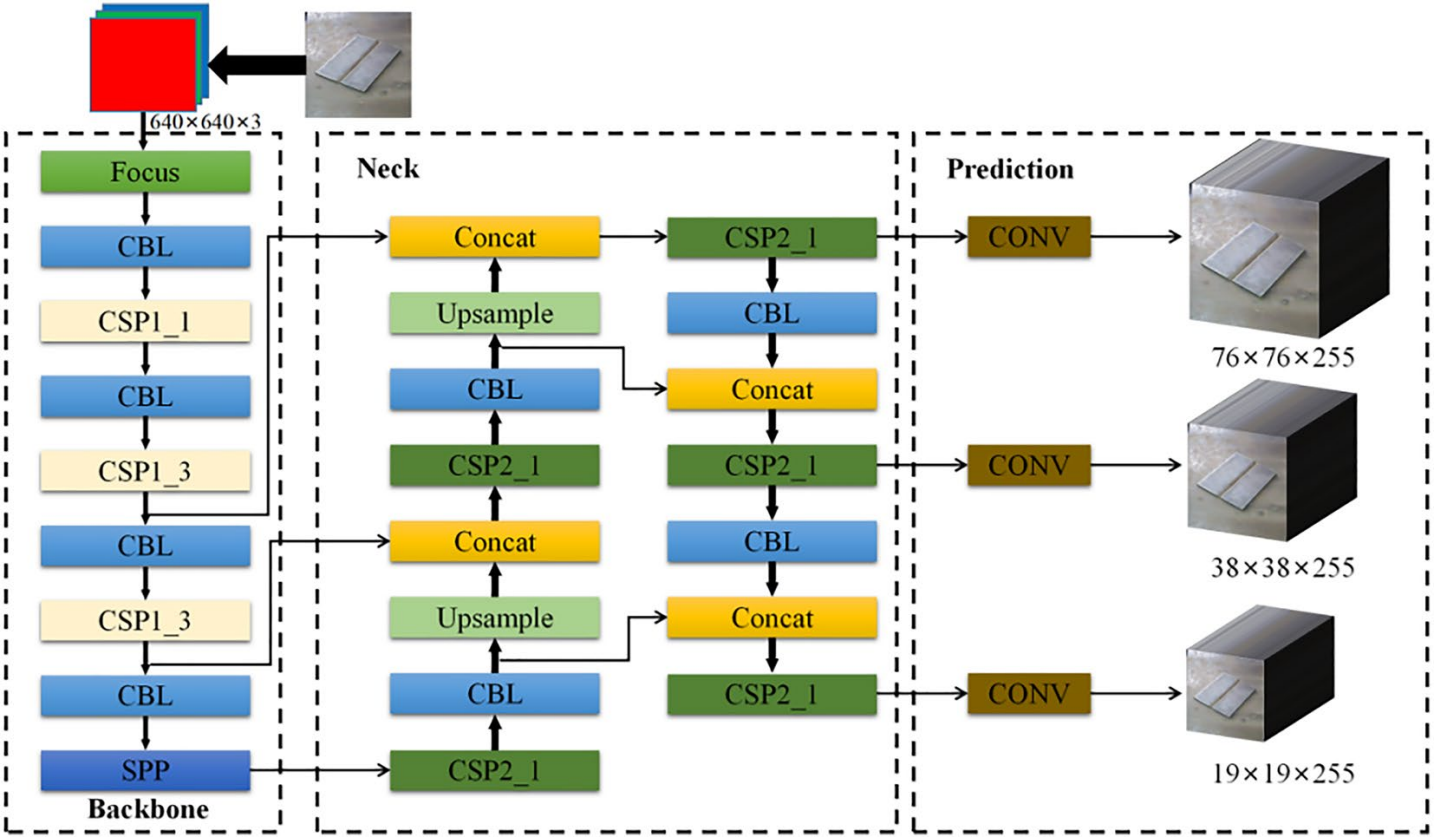
YOLOv5s network structure. Note: The dashed boxes indicate the three parts of the algorithm: backbone, neck, and prediction.

In the officially released YOLOv5 code, the detection network is divided into four versions, namely, YOLOv5x, YOLOv5l, YOLOv5m and YOLOv5s. Among them, YOLOv5s is the network with the smallest depth and feature map width, while the other three versions can be regarded as deepening and widening based on YOLOv5s. In this paper, we introduce the most basic YOLOv5s as the representative, and Fig. 2 shows the network structure of YOLOv5s.

**Figure 3:**
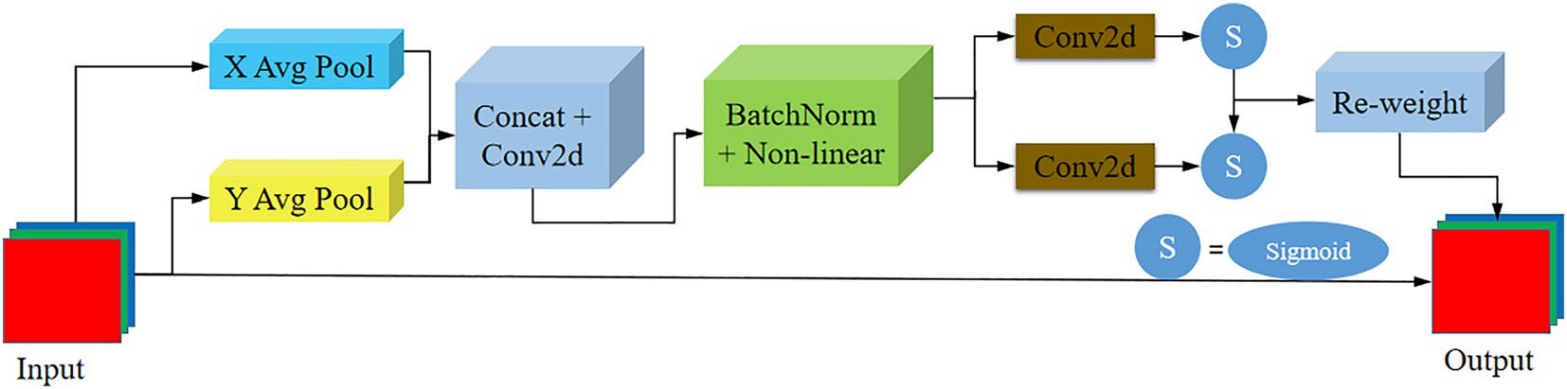
Coordinate attention module.

With an eye-in-hand calibration, the detection object and the camera are in relative motion, and there is a significant transformation in the image scale, which can cause difficulties in distinguishing the object's features. In addition, the background at the welding site is usually cluttered, and the object occupies a small area in the entire image; thus, the object has a weak presence and is easily missed. In recent years, an attention mechanism module has been widely used in computer vision tasks and can enhance the extraction of useful features to improve model feature extraction^29^. This study improves the original YOLOv5 model by adding an attention mechanism. More attention resources are devoted to weld groove feature detection, which increases the ability of the model to detect weld grooves in the cluttered background of a welding site. Introducing an attention mechanism increases the model's computational load and the computational burden. Therefore, simple and lightweight coordinate attention (CA)^30^ with little additional computational effort is utilized in this study to enhance the extraction of weld grooves in complex environments. A flow chart of the CA module is shown in Fig. 3.

Embedding the location information into the channel attention in the CA module involves two main steps: coordinate information embedding and coordinate attention generation.

Input feature map X is the output of the previous convolution layer with dimensions *C* × *H* × *W*, which means the number of channels is *C*, the height is *H*, and the width is *W*. The average pooling of dimensions *(H,1)* and *(1,W)* is used to encode each channel along the horizontal and vertical coordinate directions, that is, the output of the $c$ th channel with height $h$ and width $w$, as expressed by Eqs. (1) and (2).

1 $$ z_{c}^{h} (h) = \frac{1}{W}\sum\limits_{0 < i < W} {x_{c} } (h,i) $$

2 $$ z_{c}^{w} (w) = \frac{1}{H}\sum\limits_{0 \le j < H} {x_{c} \left( {j,w} \right)} $$

where $h$ and $w$ are the height and width of the input feature map corresponding to the current attention module, $z_{c}^{h} (h)$ denotes the output of the $c$ th channel with height $h$, and $z_{c}^{w} (w)$ denotes the output of the $c$ th channel with width $w$.

Equations (1) and (2) are aggregate features along the two spatial directions. The two cascaded feature maps $z^{h}$ and $z^{w}$ are generated. Then, convolution operation $F_{1}$ with a convolution kernel size of 1 is performed to generate an intermediate feature map $f$ for the spatial information in the horizontal and vertical directions. This is expressed by Eq. (3).

3 $$ f = \delta \left( {F_{1} \left( {\left[ {z^{h} ,z^{w} } \right]} \right)} \right) $$

The feature map $f$ is divided into two independent tensors $f^{h}$ and $f^{w}$ along the spatial dimensions. Then, feature maps $f^{h}$ and $f^{w}$ are transformed into the same number of channels as the input *X* using two convolutional operations $F_{h}$ and $F_{w}$ with a convolutional kernel of size 1 to obtain the attention weights $g^{h}$ and $g^{w}$ in both directions. This is expressed by Eqs. (4) and (5).

4 $$ g^{h} = \sigma \left( {F_{h} \left( {f^{h} } \right)} \right) $$

5 $$ g^{w} = \sigma \left( {F_{w} \left( {f^{w} } \right)} \right) $$

where $\sigma$ is the sigmoid activation function. A range of values from 0 to 1 is obtained after the operation, which represents the degree of importance. We expand $g^{h}$ and $g^{w}$ as the attention weights, and the final output is shown as Eq. (6).

6 $$ y_{c} (i,j) = x_{c} (i,j) \times g_{c}^{h} (i) \times g_{c}^{w} (j) $$

**Figure 4:**
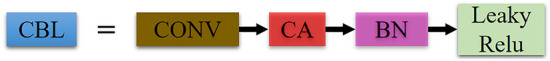
Improved CBL module.

It is possible to effectively focus on the effective channel while paying attention to the spatial location coordinate information, as shown in Fig. 4. In this study, the attention mechanism is embedded into a CBL module of YOLOv5 to increase the efficiency of the model in feature extraction for our object of interest. The CBL module is widely used in YOLOv5 to help a model better extract image features and improve the performance and accuracy of a model. For example, more attention is given to the weld grooves on the workstation, which significantly increases the efficiency of model training.

**Figure 5:**
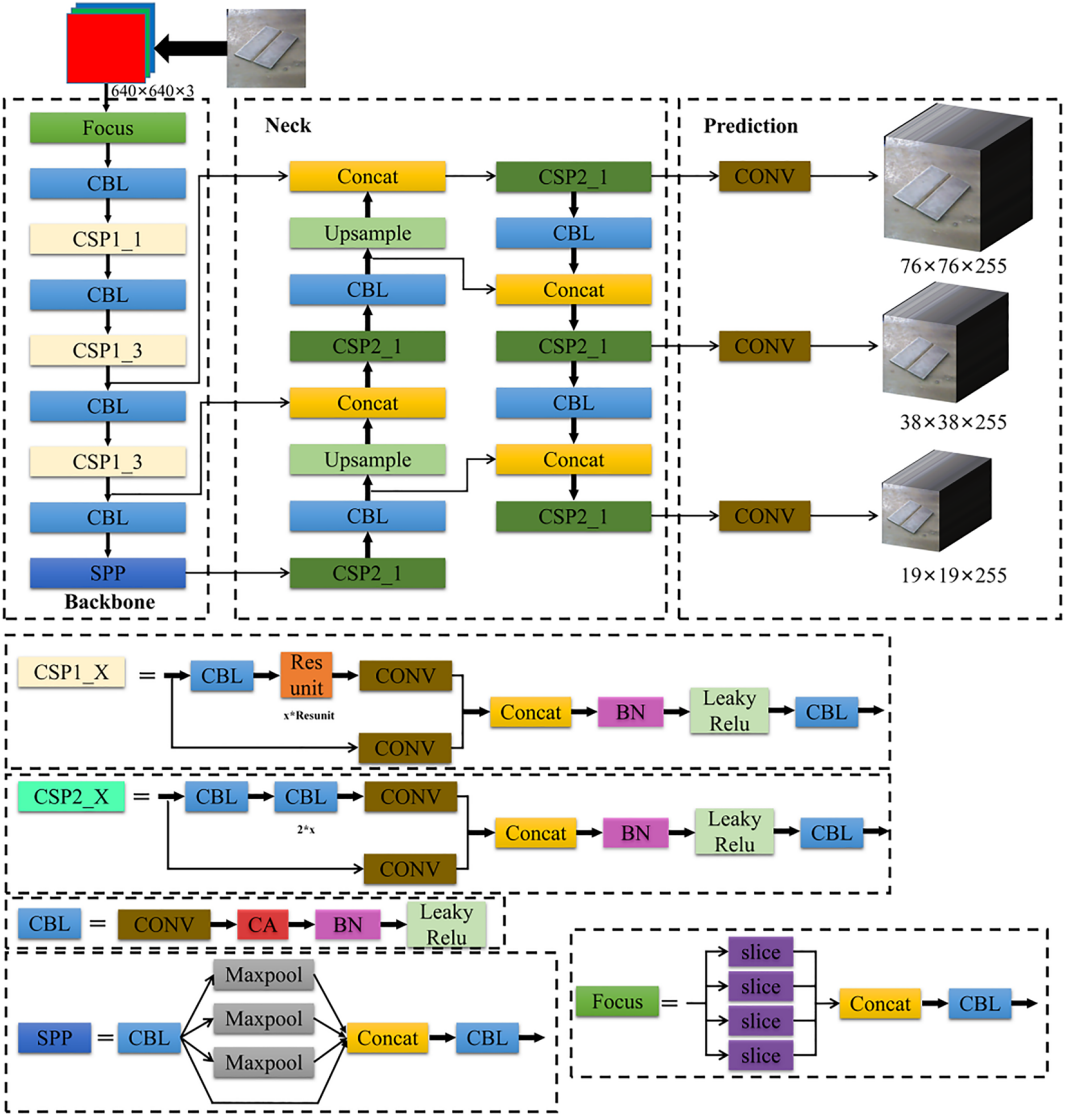
Improved YOLOv5s network structure. Note: ResUnit is the basic building block in ResNet, consisting of a residual join and two convolutional layers; Slice is the slicing operation; CONV is the normal convolution operation; Leaky ReLU is the activation function; Add is the superposition operation; BN is the batch normalization; SPP is the spatial pyramid pooling operation; Maxpool is the maximum pooling operation; Concat is the dimensional stitching operation.

Considering that most production sites have embedded devices with low computing power, the YOLOv5s network with the smallest YOLOv5 model is selected as the benchmark network for the weld groove detection algorithm on the basis of economical and practical perspectives. The framework of the improved YOLOv5 algorithm is shown in Fig. 5.

### Weld groove positioning based on a depth camera

#### Relationships among coordinate systems

**Figure 6:**
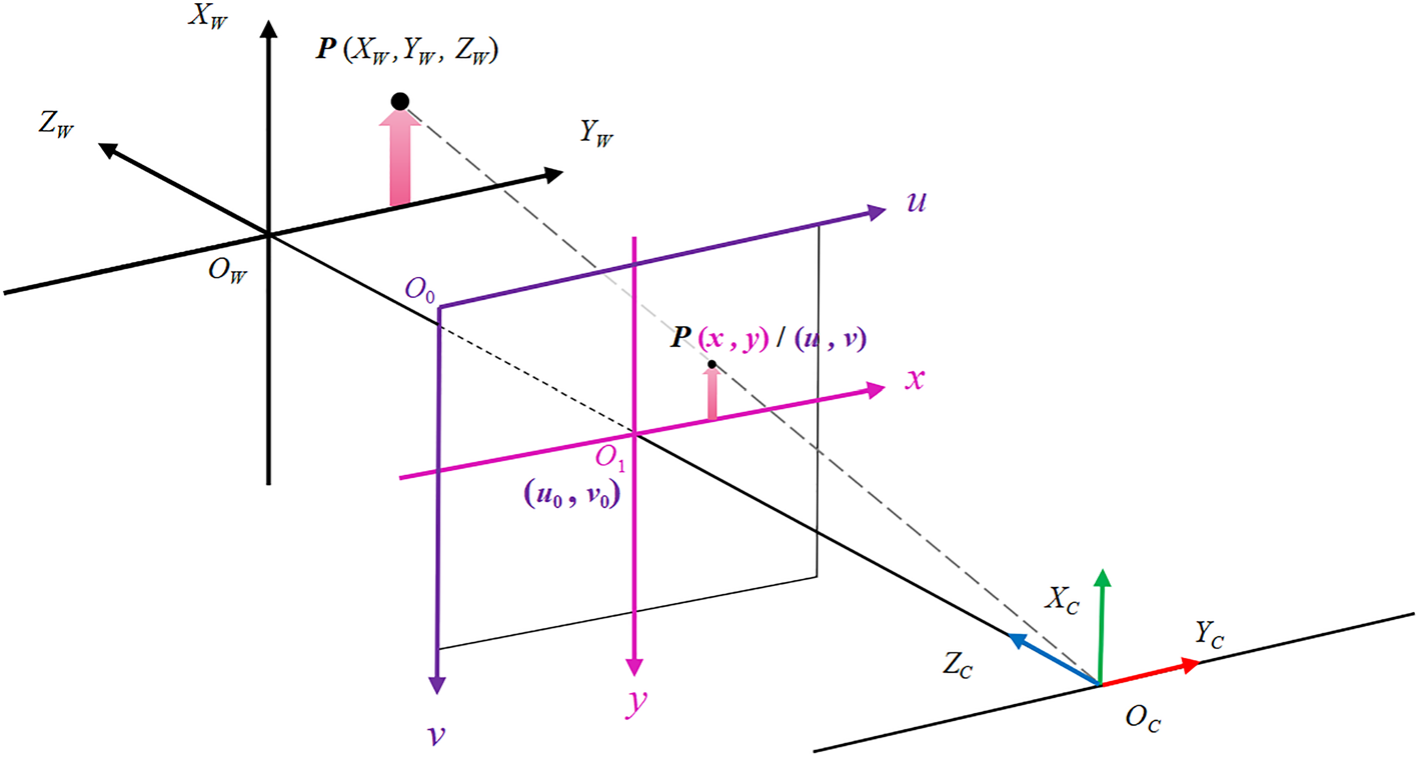
System coordinate systems and their relationships.

A geometric camera imaging process model can establish the relationship between coordinates in 3D space and the pixel coordinates of points in an image. The parameters of such a model are known as the camera parameters. Camera calibration dramatically impacts both image processing and machine vision applications. The accuracy of the calibration results often determines the accuracy of subsequent image analysis. The higher the camera calibration accuracy, the more accurate the results of the image processing are obtained. Four coordinate systems, the world coordinate system, camera coordinate system, image coordinate system, and pixel plane coordinate system, must be studied to acquire their transformation relationship. Figure 6 shows the relationships among the four coordinate systems.

The world coordinate system ($O_{W} - X_{W} Y_{W} Z_{W}$) is the reference coordinate system in positioning systems. Any point in space can be found and described in the world coordinate system. Thus, the interrelationship between the camera and other objects in space can be established.

The camera coordinate system ($O_{C} - X_{C} Y_{C} Z_{C}$) usually takes the optical center as the coordinate origin. The $X_{C}$ and $Y_{C}$ axes in the camera in space and the image $x$ and $y$ axes are parallel. According to the right-hand rule, the $Z_{C}$ axis can be determined, and the direction is always perpendicular to the image plane. The transformation of the world coordinate system from the camera coordinate system is a rigid body transformation, which can be converted by a simple rotation transformation R and translation transformation T.

The image coordinate system ($O_{1} - xy$) is located on the imaging plane of the camera, and the intersection of $Z_{C}$ and the imaging plane in the camera coordinate system is regarded as the coordinate origin. The $x$ and $y$ axes in the imaging plane are perpendicular to each other.

The pixel coordinate system ($O_{0} - uv$) is also located on the imaging plane with the coordinate origin $O_{0}$ in the upper left corner of the image. The $u$ and $v$ axes are also perpendicular to each other and parallel to the $x$ and $y$ axes in the image coordinate system.

The relationship between the world coordinate system and the pixel coordinate system can be expressed by Eq. (7).

7 $$ \begin{aligned}   Z_{c} \left[ {\begin{array}{*{20}c}    u  \\    v  \\    1  \\   \end{array} } \right] &  = \left[ {\begin{array}{*{20}l}    {f_{x} } \hfill & 0 \hfill & {u_{0} } \hfill & 0 \hfill  \\    0 \hfill & {f_{y} } \hfill & {v_{0} } \hfill & 0 \hfill  \\    0 \hfill & 0 \hfill & 1 \hfill & 0 \hfill  \\   \end{array} } \right]\left[ {\begin{array}{*{20}c}    {\mathbf{R}} & {\mathbf{t}}  \\    {\mathbf{0}} & 1  \\   \end{array} } \right]\left[ {\begin{array}{*{20}c}    {X_{w} }  \\    {Y_{w} }  \\    {Z_{w} }  \\    1  \\   \end{array} } \right] \\     &  = K_{1} K_{2} \left[ {\begin{array}{*{20}c}    {X_{w} }  \\    {Y_{w} }  \\    {Z_{w} }  \\    1  \\   \end{array} } \right] \\  \end{aligned}  $$

where $K_{1}$ is the intrinsic camera property and $K_{2}$ is the extrinsic camera property.

That is, if the intrinsic and extrinsic camera properties are known, the real-world coordinates can be obtained from the pixel positions in the image.

#### Camera parameter calibration

**Figure 7:**
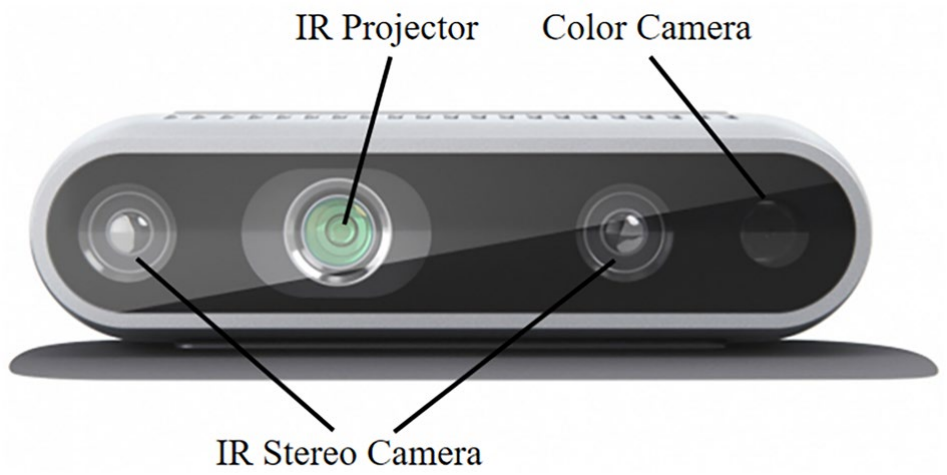
Intel RealSense D435i.

A RealSense D435i depth camera is used to obtain the real coordinates of the weld groove. The camera operation is based on the principle of triangulation, and IR stereo cameras located on the left and right sides measure the weld groove depth. An infrared point projector on the left can improve depth measurement accuracy in some cases where the texture is not apparent. An RGB color camera on the right captures color pictures. The Intel RealSense D435i camera is shown in Fig. 7.

**Figure 8:**
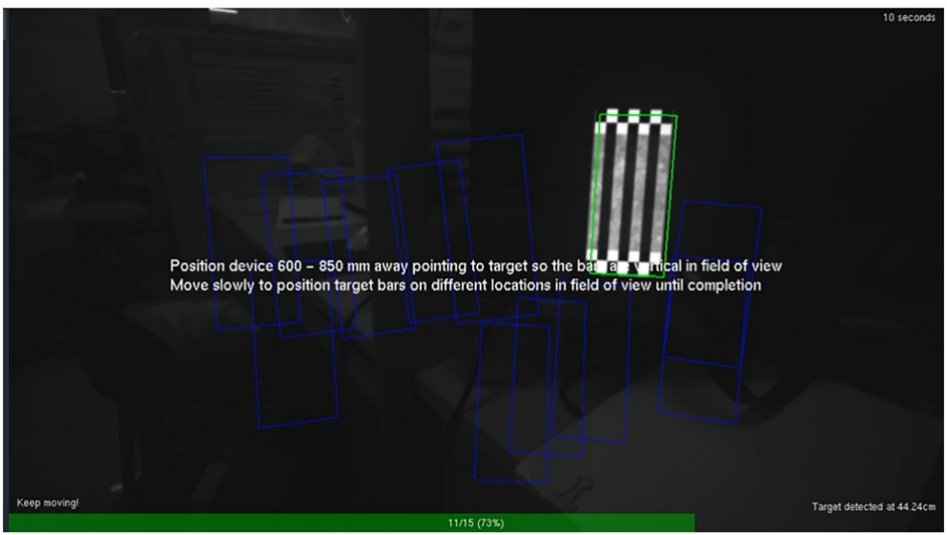
Camera calibration.

The official Intel RealSense Dynamic Calibrator software and the camera calibration board provided by Intel and the official included camera calibration board are used to calibrate the camera. With these methods and software, the intrinsic and extrinsic parameters of the depth camera and RGB camera can be quickly obtained, and the cameras can be calibrated at the same time. After calibration, the calibration results replace the precalibration parameters to obtain more accurate results. The calibration board used can be accessed with cell phone software, making this calibration process easy and fast for outdoor work environments. The camera calibration process is shown in Fig. 8, and the calibration results are shown in Table 1.

**Table 1:** Depth camera calibration results.

Intrinsic parameters	RGB camera	Depth camera
$f_{x}$	606.709	422.659
$f_{y}$	605.348	422.659
$u_{0}$	426.719	424.256
$v_{0}$	241.077	234.522

According to Table 1, the depth camera to RGB camera conversion rotation matrix is shown in Eq. (8), and the conversion translation matrix is shown in Eq. (9).

8 $$ R = \left( {\begin{array}{*{20}c} {0.999811} & { - 0.019247} & { - 0.002839} \\ {0.019251} & {0.999814} & {0.001495} \\ {0.002809} & { - 0.001549} & {0.999995} \\ \end{array} } \right) $$

9 $$ T = \left[ {\begin{array}{*{20}c} { - 0.014946} & { - 0.000028} & { - 0.000185} \\ \end{array} } \right]^{T} $$

### Improved YOLOv5 detection algorithm combined with a depth camera localization algorithm

**Figure 9:**
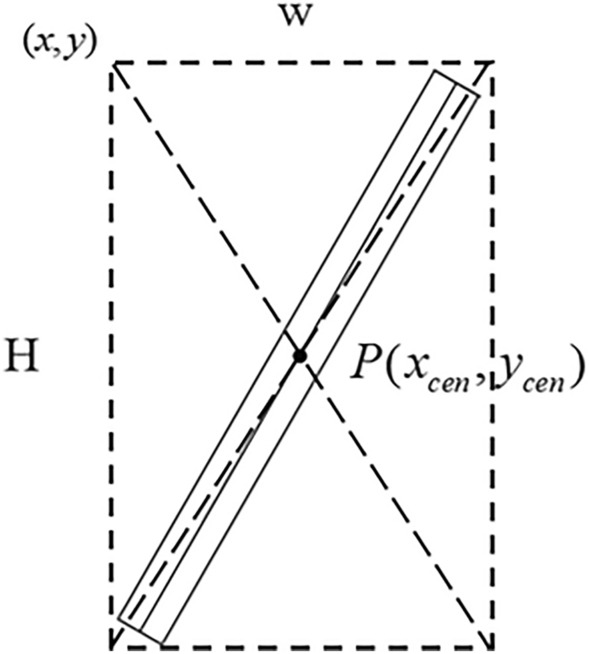
Plane location based on the prediction box detected using the improved YOLOv5.

The research object of this study is a V-groove weld plate, and the weld groove can be regarded as a 50 mm × 300 mm long rectangle. The improved YOLOv5 output obtains the weld groove prediction frame, and the weld groove must be on the diagonal center of the rectangle prediction frame. Therefore, the predicted frame center point of the weld groove obtained by the improved YOLOv5 can be used as the center coordinates of the weld groove in the plane coordinates, as shown in Fig. 9. The center point coordinates $P(x_{cen} ,y_{cen} )$ are denoted as Eq. (10).

10 $$ \begin{gathered} xcen = x + W/2 \hfill \\ y_{cen} = y + H/2 \hfill \\ \end{gathered} $$

**Figure 10:**
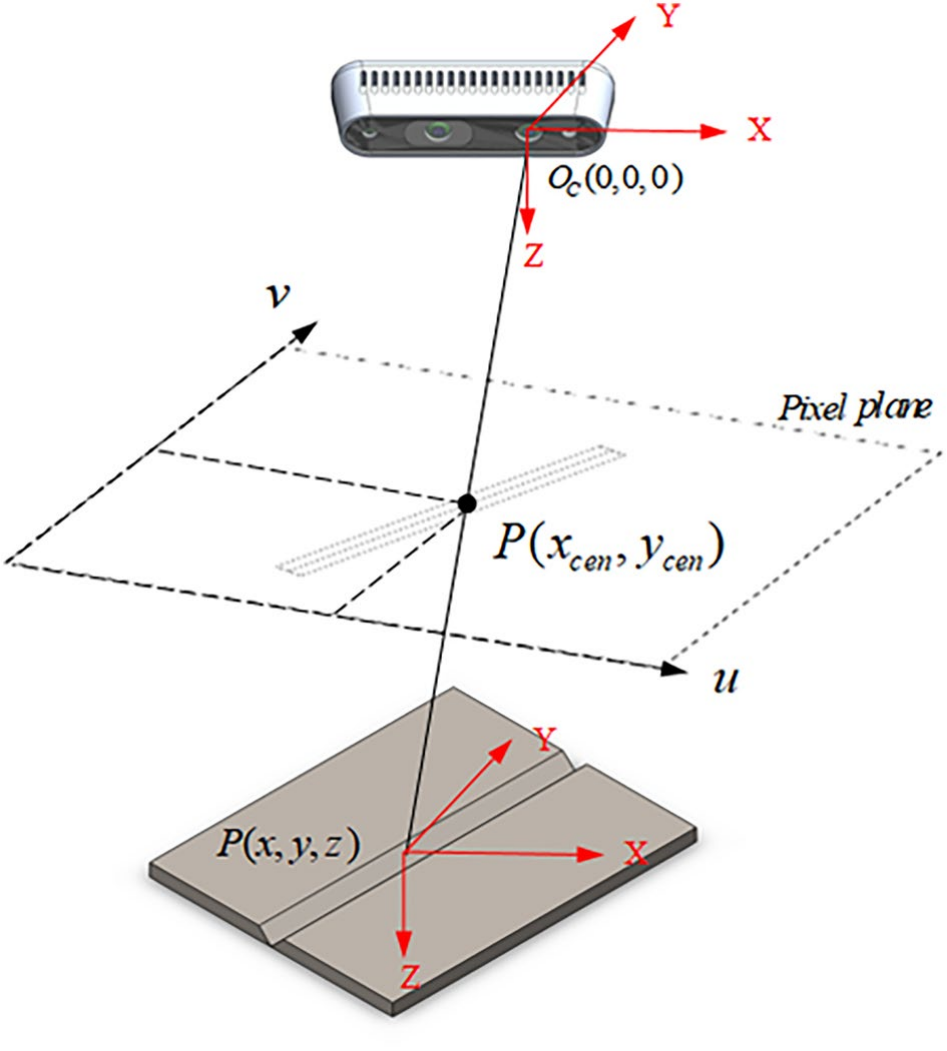
Relationship between the spatial coordinates of the weld groove center point with the pixel plane and depth camera.

The spatial relationship between the pixel plane of the weld groove and the camera is shown in Fig. 10. $P(x,y,z)$ is the spatial coordinate of the center point of the workpiece surface. The center point coordinates $P(x_{cen} ,y_{cen} )$ are analyzed using the improved YOLOv5 detection method. The RGB image is matched with the depth point cloud to obtain the pixel planes of the point cloud and thus the corresponding depths of the center points. The position of the weld groove can be converted to the spatial coordinates $P(x,y,z)$ under the camera coordinates $O_{C} (0,0,0)$ for the left lens of the camera.

The pyrealsense2 interface library of the Intel RealSense D435i camera is used in combination with the improved YOLOv5 algorithm to obtain the pixel location information of the object area. When the depth camera acquires an image, the position of the weld groove in the image is obtained using the object detection algorithm, the 3D point cloud of the weld groove is obtained using the depth sensor of the RealSense camera, and the distance of the pixel from the camera can be obtained. Thus, the 3D coordinate data of the center of the rectangular frame of the object in the camera coordinate system can be obtained.

## Experiment and analysis

### Improved YOLOv5 performance evaluation

The performance of the improved algorithm is compared with the implementation of the previously improved algorithm to verify the accuracy and real-time performance of the method. Through image acquisition and image processing, a V-shaped weld groove dataset was obtained. The dataset was tested on the test platform built to compare the algorithm's performance before and after the improvement.

#### Datasets and processing platforms

**Figure 11:**
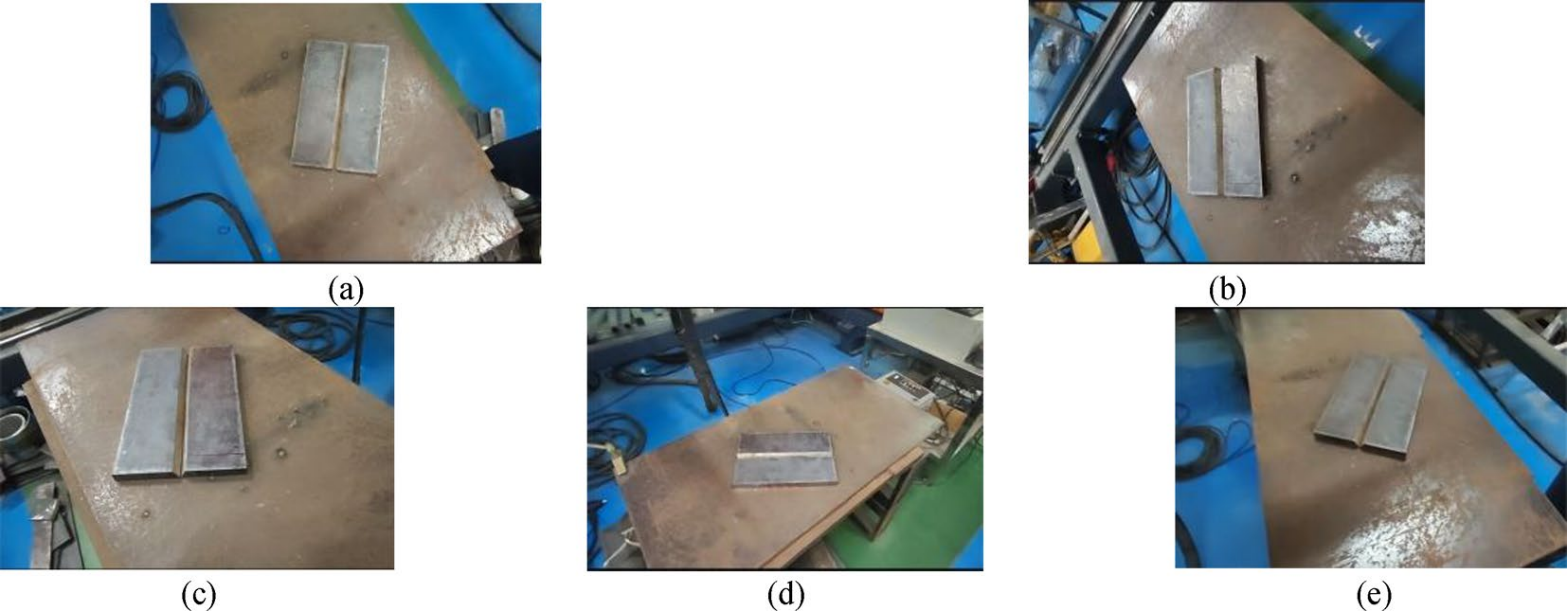
Weld groove images from different visual angles of the workpiece. Note: (**a**) directly above; (**b**) 45° to the right; (**c**) 45° to the front; (**d**) 45° to the left; (**e**) 45° to the back.

V-groove plates were selected as the subjects of this study. To expedite model training and enhance recognition efficiency, all images were acquired using the Intel RealSense D435i camera, with consistent image dimensions of 1280 × 720 pixels. A total of 800 images of V-groove plates, taken from various angles, were collected to showcase the diverse impact of different working conditions on weld groove identification. Among these, a total of 800 images were captured, covering various angles, including directly above, 45° in front, 45° to the left, 45° to the right, and 45° to the back, as illustrated in Fig. 11 for more details.

**Figure 12:**
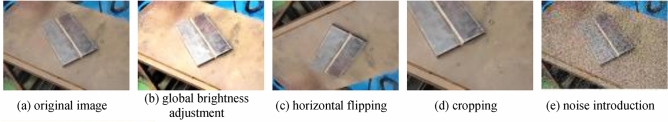
Image augmentation effect.

To augment the dataset and enhance the model's generalization performance, this study employed four image augmentation techniques: global brightness adjustment, horizontal flipping, cropping, and noise introduction. The effects are illustrated in Fig. 12. These operations expanded the initial 800 images to a total of 4000 images.

The 4000 images were randomly divided into training and validation sets at a 7:3 ratio, resulting in 2800 images in the training set and 1200 images in the validation set. Detailed information about the datasets post-augmentation can be found in Table 2.

**Table 2:** Datasets after data augmentation.

Datasets	Directly above	45° to the right	45° to the front	45° to the left	45° to the back	Total
Before enhancement	164	210	196	106	124	800
After enhancement	820	1050	980	530	620	4000
Training set	574	735	686	371	434	2800
Validation set	246	315	294	159	186	1200

On the hardware platform with a Windows 10 operating system, Intel i7-12700 CPU, 32 GB RAM, and NVIDIA RTX3070 GPU, the dataset was trained using the proposed improved YOLOv5 algorithm. Due to memory limitations, the batch size was set to 8. The momentum, learning rate, and weight decay parameters were the default YOLOv5 parameters and were set to 0.937, 0.001, and 0.0005, respectively.

#### Evaluation indicators

Network performance is evaluated using evaluation indicators for deep learning object detection, such as *Precision*, *mAP*, and *Recall* . The *Recall* is denoted by Eq. (11)

11 $$ Recall \, = \frac{TP}{{TP + FN}} $$

The *Precision* is denoted by Eq. (12)

12 $$ Precision \, = \frac{TP}{{TP + FP}} $$

where *TP* is the number of correct weld groove detections, *FN* is the number of missed weld grooves, and *FP* is the number of false alarm weld grooves.

The *mAP* is denoted by Eq. (13)

13 $$ mAP = \frac{{\sum\nolimits_{n = 1}^{N} A P_{n} }}{N} $$

where *N* is the number of object types, *n* is the *n*th type, and *AP* denotes the detection accuracy of the *n*th type of object. In this study, the intersection over union (IoU) is set to 0.5, meaning that the inspection frame is considered correct when the overlap area between the predicted frame and the real frame is greater than 50%.

### Performance evaluation and comparative analysis

In this study, we conducted a comprehensive evaluation of our proposed method, comparing it with several state-of-the-art models, including Faster R-CNN, DETR, and YOLO-V5. Additionally, we analyzed the performance of our 'Proposed method' based on key parameters, including mAP (mean average precision), training epochs, and model size (Model-size/M). The detailed results of these comparisons can be found in Table 3.

**Table 3:** Experimental comparisons of different methods.

Method	mAP (%)	FPS	Epochs	Model-size/M
Faster R-CNN	83.2	5	250	359.2
DETR	85.3	6	500	463.8
YOLO-V5	82.3	23	250	15.3
Proposed method	90.8	20	250	17.2

The mAP values of the improved YOLOv5 network show significant enhancements compared to those of the YOLOv5 network, demonstrating that the improved network offers increased accuracy, reliability, and overall superior performance. While the improved YOLOv5 algorithm enhances detection performance, it's essential to note that the increased network structure parameters do impact the network's computational speed.To assess whether the improved network aligns with the system's performance requirements concerning testing speed, we deployed the trained model on a host computer equipped with a GTX960M GPU for real-time weld groove detection using RealSense cameras. The results of the detection frame rate tests are also illustrated in Table 3.

Firstly, in terms of the mAP metric, our proposed method achieved an impressive mAP score of 90.8% in the welding seam recognition task. This is a significant performance advantage over other methods such as Faster R-CNN (83.2%) and DETR (85.3%), indicating that our method can accurately locate welding seams with higher precision, providing more reliable guidance for welding tasks. Before the YOLOv5 algorithm improvement, the mAP was 82.3%, and after the algorithm improvement, the mAP reached 90.8%, which is a significant improvement. The attention mechanism module has a more significant impact on weld groove detection, primarily because the original algorithm is less clear about the extraction of features and can be disturbed by a complex background. Moreover, the object features change considerably when the camera is moving, causing missed detection. The model with the CA module is more accurate in extracting salient features of weld grooves.

Secondly, real-time performance holds significant importance in various welding applications. In terms of FPS, our method has demonstrated exceptional real-time capabilities, achieving a rate of 20 FPS, which is comparable to YOLO-V5 (23 FPS). When compared to the slower processing speeds of Faster R-CNN (5 FPS) and DETR (6 FPS), our method clearly meets the real-time demands of welding tasks effectively. The improved YOLOv5 network still achieves an average detection frame rate of 20 FPS. This performance comfortably aligns with the requirements for weld groove detection in practical production scenarios.

Furthermore, it is noteworthy that our method boasts a model size advantage. With a model size of 17.2 MB, it is significantly smaller than the model size of DETR (463.8 MB). This is particularly advantageous for deployment in embedded systems or resource-constrained environments, making model deployment more convenient. Lastly, our method exhibits excellent performance despite requiring fewer training epochs compared to other methods. This suggests that our method converges faster and is operationally efficient in real-world applications.

In conclusion, based on these experimental results, we conclude that our proposed welding seam recognition method offers significant advantages in terms of accuracy, real-time capability, and model size, providing robust support for welding automation and precision.

### Experiment on weld groove identification and guiding

**Figure 13:**
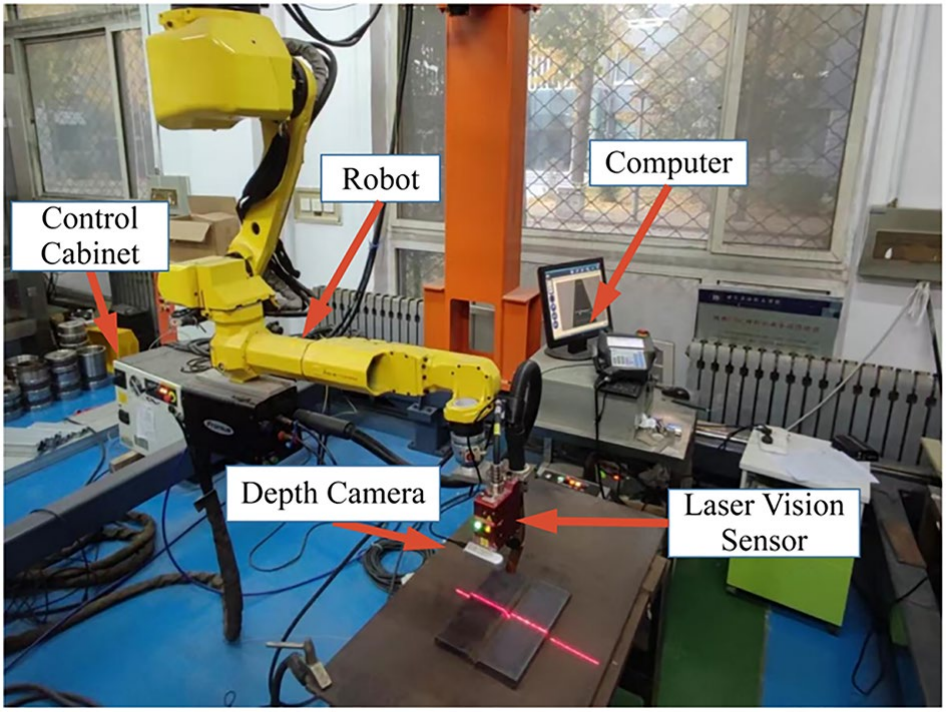
Experimental platform for the robot welding guidance system.

To verify the algorithm, it was deployed to the robot host computer, the camera was installed on the robot, and V-shaped weld grooves on a workstation were identified and positioned at different distances as a test experiment. The test hardware included an Intel RealSense D435i depth camera, a Servo-Robot laser vision sensor, a Fanuc robot with its control cabinet, and a host computer. The host computer included 8 GB memory, a 4 GB GTX 960 GPU, and an Intel Core i7-6700Q. It could read and write the coordinate positions in real time through socket communication with the control cabinet. The LVS and depth camera were mounted at the end of the welding robot, creating an eye-in-hand system with the camera and robot. The robot acted as a task actuator, accepting the coordinate information transmitted from the control cabinet and moving to the specified position. The experimental platform of the robot welding guidance system is shown in Fig. 13.

**Figure 14:**
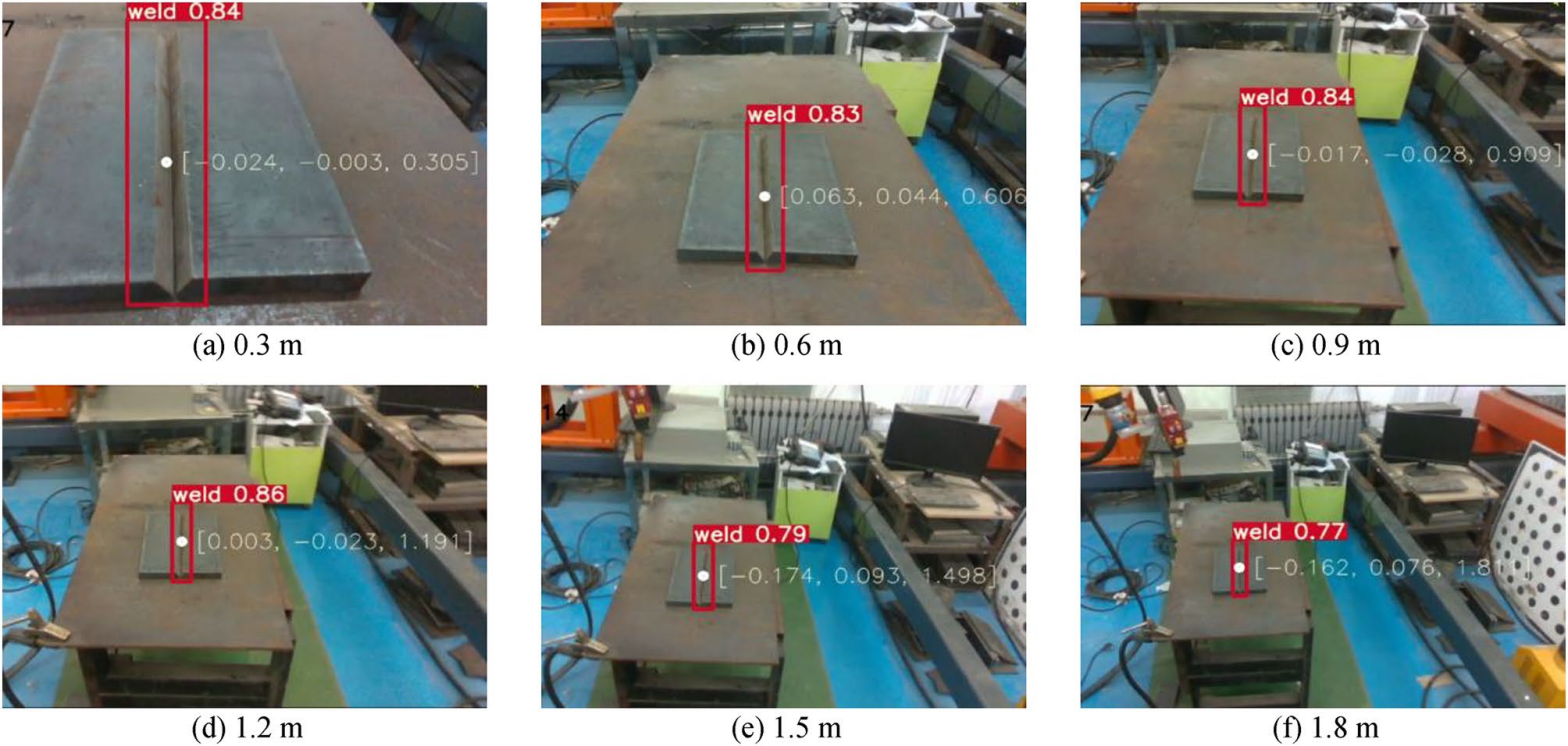
Improved YOLOv5 model weld groove detection from 0.3 to 1.8 m.

The 3D coordinates of the object position under the robot coordinate system were measured with the end of the welding gun. Its coordinates under the camera coordinate system were obtained via the hand-eye conversion matrix to obtain the depth of calculation. The depth of an object identified by the depth camera was obtained by comparing the calculated depth with the experimental depth, and the absolute error of the experimental data was received. The experiment carried out weld groove identification and guidance experiments from 0.3 to 1.8 m. The detection speed of the algorithm on the test platform reached 20 FPS, detecting and positioning the weld groove in real time. Several tests were conducted to measure the accuracy of weld groove guidance. The weld groove identification and positioning test are depicted in Fig. 14.

**Figure 15:**
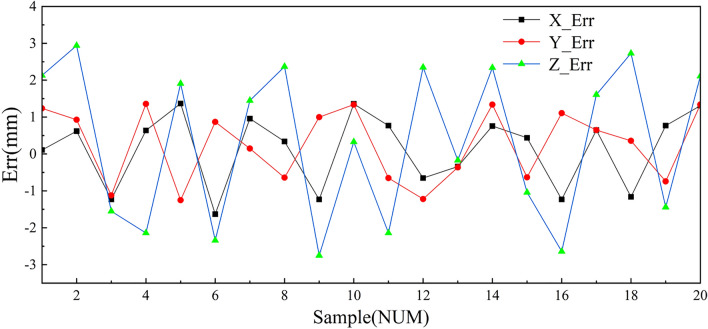
Guiding experiment error.

Twenty sets of valid experiments were conducted with a camera-to-target distance of 300 mm, and the experimental error results are given in Fig. 15. The absolute error is denoted by $AE = \sum\nolimits_{i = 1}^{n} {\left| {\Delta e_{i} } \right|}$, where $\Delta e_{i} = X_{i} - X_{0}$, $\Delta e_{i} = Y_{i} - Y_{0}$ and $\Delta e_{i} = Z_{i} - Z_{0}$. $\left( {X_{i} ,Y_{i} ,Z_{i} } \right)$ indicates the coordinates of the position of the welding torch tip and $\left( {X_{0} ,Y_{0} ,Z_{0} } \right)$ indicates the coordinates of the real position.

The absolute errors in both the X and Y directions are basically within a range of 2 mm, and the absolute errors in the Z direction are within 3 mm.

**Figure 16:**
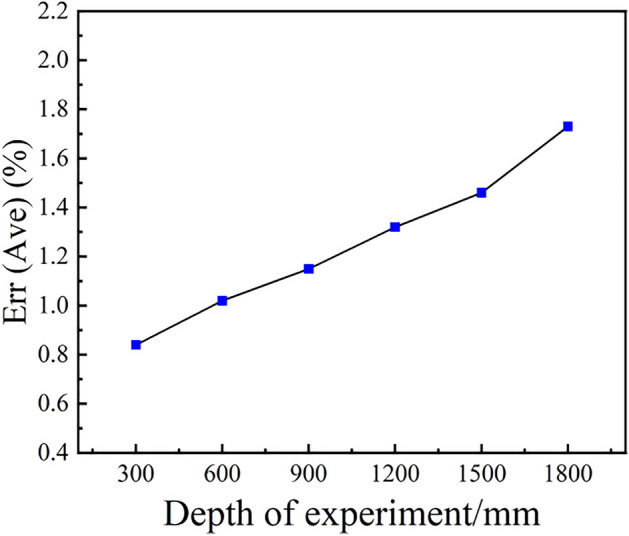
Guiding error percentage at different distances.

**Figure 17:**
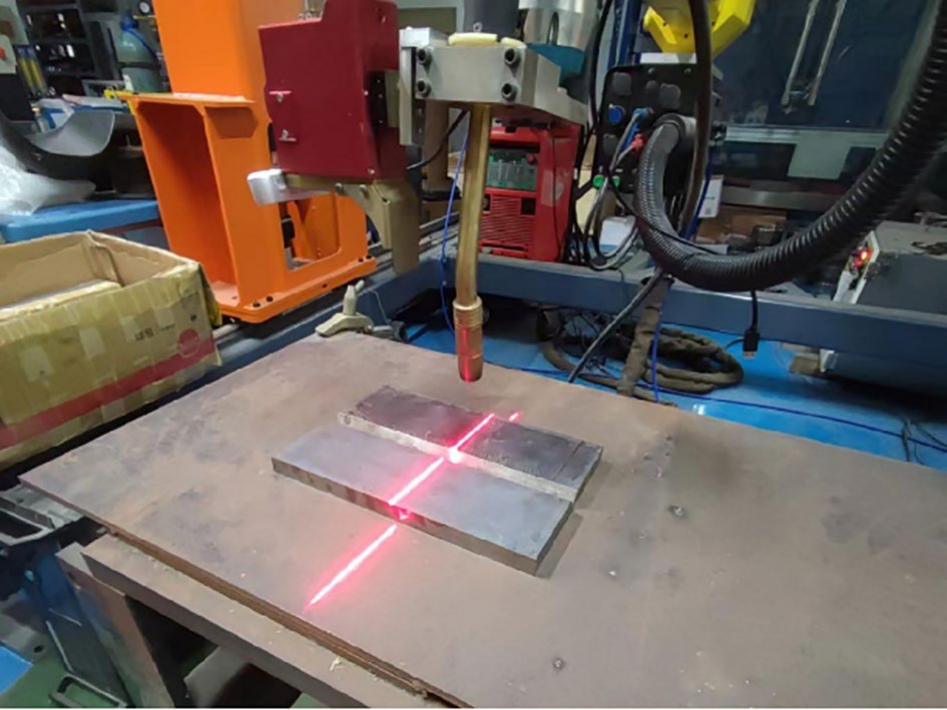
The welding torch approaches the workpiece at the specified position.

The absolute error percentage is defined as $AEP = \frac{AE}{d} \times 100\%$, where* d* represents the distance from the camera to the object to be detected. The guidance error percentage at various distances is shown in Fig. 16. The error increases with increasing distance, but the error percentage can be well effectively controlled within 2%. When the object is identified at a considerable distance, the coordinate data can be updated in real time as the camera moves to the object. By updating the position information in the position register, the additional error caused by long-distance identification can be circumvented, so the final positioning error can be controlled within 3 mm. For this system application scenario, the 3 mm error will not affect the subsequent work of the weld tracking sensor. Figure 17 shows the robot identifying the weld groove and guiding and controlling the end of the torch installed on the end of the robot to move to the specified position.

## Conclusions

To address the problem of having to manually move a weld seam tracking sensor above the weld seam before operation, a robot welding guidance system based on an improved YOLOv5 algorithm with a RealSense depth camera was proposed. The following conclusions were obtained.

1. The YOLOv5 object detection algorithm is improved by inserting a CA mechanism to achieve real-time detection of V-weld grooves in a random environment, providing an optional solution for object detection tasks on devices with limited computational resources.
2. Combining the improved object detection algorithm with a depth camera positioning algorithm, the spatial position of V-weld grooves is determined based on the object detection results using RGB images and 3D point clouds captured by a RealSense depth camera.
3. The fusion algorithm is deployed to a host computer to guide the welding robot, eliminating the reliance of an LVS on manually preset scanning trajectories. Experiments showed that the system improves the automation and intelligence of a welding robot vision system while ensuring accuracy.

In future research, the identification and positioning of more types of welds, not just V-weld grooves, will be considered. In addition, the network weight will be considered to enable the network to be deployed on platforms that require even more limited computational resources.

## Data Availability

The data essential for reproducing and verifying the findings presented in our study are sourced from third party agency. However, the accessibility of these data is subject to specific restrictions due to the terms of the license agreement under which they were obtained for the purpose of our research. Consequently, these data are not publicly available for direct download or open access.Also, corresponding author should be contacted if someone wants to request the data from this study.
